# A108 A RETROSPECTIVE REVIEW OF COMPLEX POLYP MANAGEMENT IN THE CHAMPLAIN REGION, ONTARIO

**DOI:** 10.1093/jcag/gwab049.107

**Published:** 2022-02-21

**Authors:** A Monod, A Rostom, C Dube

**Affiliations:** 1 ^1.^ University of Ottawa, Ottawa, ON, Canada; 2 ^2.^ medicine, the ottawa hospital, Ottawa, ON, Canada; 3 ^3.^ University of Ottawa, Ottawa, ON, Canada

## Abstract

**Background:**

Advanced endoscopic techniques have enabled the removal of polyps which, due to their size, location, or morphology, would otherwise have been removed surgically. Patients with such complex polyps should be referred promptly to expert centres for adjudication and management.

**Aims:**

To review patterns of referral and initial management of complex polyps at The Ottawa Hospital (TOH), to identify gaps in care and to identify strategies to address those gaps.

**Methods:**

We performed a retrospective chart review of cases where large (>3cm) colonic polyps were evaluated at TOH, from March 2019-March 2021. Cases were identified using Canadian Institute of Health Information codes. Descriptive statistics were analyzed, and were compared using Mann-Whitney U tests where appropriate.

**Results:**

94 consecutive patients with large polyps (mean age 68; 56.4% male) were included. The average polyp size was 4.7(4.4–5.1)cm. 45 patients were referred for a known complex polyp while the rest were referred for FIT/FOBT+ (n=20), symptoms or anemia (n=17), surveillance (n=9), or abnormal imaging (n=3). 32 patients were referred from >50km from Ottawa (travelling), and 62 were referred from <50km from Ottawa (local). Of the 45 referrals for known polyp, 20 (44.4%) included photodocumentation of the lesion; 33 (73.3%) had a prior biopsy, and 8 (17.8%) were partially removed. Key statistics are summarized in Table 1. All 8 patients referred with a partially removed polyp eventually had a successful endoscopic polypectomy. 10/94 (10.6%) patients required surgical resection: 7 for malignant pathology, and 3 for endoscopic failure. Overall, 3 patients had post-polypectomy bleeding, and 1 patient sustained a full-thickness perforation. As of October 2021, 26/78 (33.3%) patients eligible for a 4–6 month recheck and 36/48 (75%) patients eligible for a 12-month follow-up colonoscopy were overdue.

**Conclusions:**

This retrospective review highlights significant differences in the management of complex polyps based on patient geography and the need to develop strategies to improve access for patients referred from outside of Ottawa. As expected, COVID had a significant impact on the time to complete removal of polyps, and affected the timely endoscopic follow up of these high-risk patients. Strategies to ensure timely endoscopic follow up of patients after complex polyp removal are needed.

**Funding Agencies:**

None

**Table:** Table 1. Number of procedures and time to complete polyp removal based on patient geography, prior polyp management and COVID lockdown

	Mean # Procedures to Complete Removal	p=	Mean # Removal Attempts	p=	Time from Detection to Complete Removal (days)	p=
All Cases (n=94)	2.07		1.19		98	
Travelling Pts (n=32)	2.48	<0.01	1.44	0.036	127	0.022
Local Pts (n=62)	1.98	1.07	94
Travelling Pts Known Polyp w/ (n=22)	2.64	<0.01	1.50	<0.01	138	NS
Local Pts w/ Known Polyp (n=23)	2.35	1.22	98
Partially Removed Polyp (n=8)	2.75	NS				
Known Polyp, Not Partially Removed (n=37)	2.41				
Pre-COVID Lockdown (n=29)					86	0.013
During COVID Lockdown (n=58)					115

